# Illinois State Medical Society

**Published:** 1864-05

**Authors:** 


### ILLINOIS STATE MEDICAL SOCIETY.

The State Medical Society convened in its 12th annual session in the Common Council chamber in this city, May 3d, at 10 o'clock, A. M. In the absence of the President, Dr. A. H.

Luce, of Bloomington, 1st Vice President, called the meeting to order. Dr. N. S. Davis, Secretary.

The following members were present during the meeting:

*Cook Co.*—Drs. G. C. Paoli, R. C. Hamill, M. O. Heydock, E. L. Holmes, S. Wickersham, E. Andrews, Ira Hatch, DeLaskie Miller, Thos. Bevan, C. G. Smith, Ephraim Ingals, N. S. Davis, J. Adams Allen, W. H. Byford, R. N. Isham, J. H. Hollister, J. Bartlett, A. Fisher, J. S. Jewell, S. C. Blake, A. Groesbeck, H. M. Lyman, H. Wing.

*Edgar Co.*—Dr. J. M. Steele.

*Kane Co.*—Dr. D. W. Young.

*LaSalle Co.*—Dr. E. P. Cook.

*McLean Co.*—Drs. H. Noble, A. H. Luce.

*Morgan Co.*—Drs. D. Prince, R. E. McVey.

*Iroquois Co.*—Dr. L. F. Hewins.

*Members by Invitation.*—Drs. Mascom, of Iowa; S. H. Holden, U. S. A. The Treasurer's Report showed balance due printer for publishing Transactions for 1863, $53.

The following committee was appointed to nominate officers for the ensuing year, viz., Drs. R. C. Hamill, R. E. McVey: H. Noble, E. P. Cook, J. M. Steele, G. S. Whitmire, L. T. Harris, D. W. Young.

The annual assessment upon the members was fixed at $3.

On motion of Dr. Miller, the meeting adjourned to 2, P. M.

AFTERNOON SESSION.

The meeting was called to order by Dr. Luce, Vice President. The committee on nominations reported the following names for officers for the ensuing year:

*President*—A. H. Luce, M. D., of Bloomington.

1*st Vice President*—J. H. Steele, M. D., of Grandview.

2*d Vice President*—Thos. Bevan, M. D., of Chicago.

*Treasurer*—J. H. Hollister, M. D., of Chicago.

COMMITTEES: *Practical Medicine*—Drs. J. Adams Allen, Chairman, C. Goodbrake, H. Noble.

*Drugs and Medicines*—Drs. J. Bartlett, F. R. Payne, T. D. Fitch.

*Obstetrics*—Drs. W. H. Byford, L. Clark, S. F. Trowbridge.

*Snrgery*—Drs. A. L. McArthur, D.W. Young, L. F. Hewins.

The committee also recommended Bloomington as the place for holding the next meeting of the Society. On motion, the report was adopted, and the officers named declared duly elected. A letter was read from Dr. A. McFarland, President, regretting his unavoidable absence.

Dr. R. E. McVey read an interesting paper on Cerebro-Spinal Meningitis. Dr. J. Adams Allen read a portion of an able and interesting paper on the same subject. Dr. J. S. Whitmire made a verbal report of three cases occurring in his practice.

The Chairman of the committee on Surgery read an abstract of their annual report. Referred to the Publishing committee.

Dr. E. L. Holmes read an elaborate report on the Diseases of the Eye, which was accepted, and referred to the Publishing committee. On motion, the Society adjourned to 9½ o'clk., Wednesday morning.

SECOND DAY—WEDNESDAY, MAY 4th.

The Society met pursuant to adjournment, the President in the chair. Minutes of the preceding day read and approved.

Drs. N, F. Burdick and Joseph Tefft, of Elgin, were elected members of the Society.

Dr. Miller, of Chicago, Chairman of the committee on Obstetrics, presented a report, which was accepted, and referred to the Publishing committee.

Dr. Prince, of Jacksonville, presented a report on Orthopœdic Surgery, which was accepted, and referred to Publishing committee. The meeting adjourned to 2 o'clock, P. M.

AFTERNOON SESSION.

Dr. J. A. Allen read the remaining portion of his paper on Cerebro-Spinal Meningitis, which was received and referred to the Publishing committee.

The Society then adjourned to 9½ o'clock, Thursday morning, and devoted the remaining portion of the day to visiting Rush Medical College, and the Marine Hospital.

THIRD DAY—THURSDAY, MAY 5th.

Society called to order by Dr. A. H. Luce, President. Minutes of previous meeting read and approved.

The names of James H. O'Kelley, William R. Fox, and Matthew J. Johnson were read as members.

The committee on Nominations submitted the following as *Committee of Arrangements*—Drs. O. F. Worrell, S. W. Noble, J. D. Rogers.

*Assistant Secretary*—Dr. R. C. Parke.

*Special Committee on Ophthalmy*—Drs. E. L. Holmes, R. E. McVey, J. S. Whitmire.

*Special Committee on Orthopædic Surgery*—Dr. D. Prince.

*Special Committee on Spotted Fever*—Dr. J. S. Jewell.

The report was adopted.

Drs. Bartlett, McFarland, and Wing, were appointed a committee to consider in what respects, the pecuniary interests of the Medical Profession suffer from unfavorable or deficient legislation; also to petition the Legislature in behalf of this Society, for such legislation as they may deem necessary and proper.

Appropriate resolutions on the death of Dr. Shubal York, of Paris, Edgar county, were adopted.

Dr. Young, of Aurora, offered the following, which was adopted:

*Resolved*, That the present pay and rank of Surgeons and Assistant Surgeons in the army is inadequate to compensate them for the services required of and performed by them, and

*Resolved*, That the members of this Association ought to make every possible exertion, through the National Medical Association, and our Senators and Representatives in Congress, to have our Medical brethren in the field receive at least a reasonable compensation for their services and sacrifices, while they are braving the dangers of camps, and caring for the soldiers of our country.

Dr. Wing, of Chicago, offered the following, which was adopted:

*Resolved*, That the profession is the proper judge of the qualifications of its members.

*Resolved*, That the interests of the profession will be promoted by having new members admitted on the judgment of the profession itself, and that, therefore,

*Resolved*, That the committee appointed under resolution to memorialize the State Legislature be instructed also to ask that the Society be authorized by law to grant certificates of qualification, on proper examination, to under graduates.

Dr. Davis made the annual report of the committee on Practical Medicine, which was referred to the Publishing committee.

Drs. G. B. Lester, and F. A. Emmons were elected members.

A vote of thanks to the Officers of the Society, and to the Common Council of the city of Chicago, was then adopted.

The following named gentlemen were selected as delegates to the ensuing meeting of the American Medical Association, viz., Drs. N. Wright, D. Prince, R. E. McVey, H. Noble, N. S. Davis, D. W. Young, J. M. Steele, J. Woodworth, J. Bartlett, J. S. Jewell, Lucius Clark, R. C. Hamill, N. Bottom, A. L. McArthur, E. P. Cook, T. D. Fitch, S. W. Noble, C. Goodbrake.

The Society then adjourned *sine die.*

